# Ionic Liquid-Based Polymer Matrices for Single and Dual Drug Delivery: Impact of Structural Topology on Characteristics and In Vitro Delivery Efficiency

**DOI:** 10.3390/ijms25021292

**Published:** 2024-01-20

**Authors:** Katarzyna Niesyto, Shadi Keihankhadiv, Aleksy Mazur, Anna Mielańczyk, Dorota Neugebauer

**Affiliations:** Department of Physical Chemistry and Technology of Polymers, Faculty of Chemistry, Silesian University of Technology, 44-100 Gliwice, Poland; katarzyna.niesyto@polsl.pl (K.N.); shadi.keihankhadiv@polsl.pl (S.K.); aleksy.mazur@polsl.pl (A.M.); anna.mielanczyk@polsl.pl (A.M.)

**Keywords:** co-delivery systems, single drug systems, dual drug systems, poly(ionic liquid)s, graft copolymers, linear copolymers, *p*-aminosalicylate, isoniazid

## Abstract

Previously reported amphiphilic linear and graft copolymers, derived from the ionic liquid [2-(methacryloyloxy)ethyl]trimethylammonium chloride (TMAMA_Cl‾), along with their conjugates obtained through modification either before or after polymerization with *p*-aminosalicylate anions (TMAMA_PAS‾), were employed as matrices in drug delivery systems (DDSs). Based on the counterion type in TMAMA units, they were categorized into single drug systems, manifesting as ionic polymers with chloride counterions and loaded isoniazid (ISO), and dual drug systems, featuring ISO loaded in self-assembled PAS conjugates. The amphiphilic nature of these copolymers was substantiated through the determination of the critical micelle concentration (CMC), revealing an increase in values post-ion exchange (from 0.011–0.063 mg/mL to 0.027–0.181 mg/mL). The self-assembling properties were favorable for ISO encapsulation, with drug loading content (DLC) ranging between 15 and 85% in both single and dual systems. In vitro studies indicated ISO release percentages between 16 and 61% and PAS release percentages between 20 and 98%. Basic cytotoxicity assessments using the 2,5-diphenyl-2H-tetrazolium bromide (MTT) test affirmed the non-toxicity of the studied systems toward human non-tumorigenic lung epithelial cell line (BEAS-2B) cell lines, particularly in the case of dual systems bearing both ISO and PAS simultaneously. These results confirmed the effectiveness of polymeric carriers in drug delivery, demonstrating their potential for co-delivery in combination therapy.

## 1. Introduction

The use of nanocarriers has emerged as a highly promising strategy in the field of drug delivery systems (DDSs) [1,2,3,4,5]. These systems aim to prevent harmful side effects, reduce drug degradation, enhance drug accumulation within specific target zones, and improve overall drug bioavailability [6,7,8,9,10,11]. A diverse range of drug carriers is extensively employed, such as lipoproteins, liposomes, hydrogels, conjugates, capsules, particles, as well as micellar systems [12,13,14]. Among the latter, polymeric micelles representing nano-scale dimension superstructures are formed in an aqueous environment by self-assembling amphiphilic polymers with different types, including linear and graft, block, and stimuli-sensitive [15,16,17,18]. They are notable for their considerable potential, attributed to their dual-phase arrangement, featuring an inner core and an outer shell [19,20]. The hydrophobic core of micelles offers a conducive environment for solubilizing water-insoluble drugs, enabling their effective loading and delivery to the designated targets [21,22]. Moreover, they demonstrate kinetic stability in the bloodstream due to low critical micelle concentrations (CMCs) [23], leading to prolonged circulation duration and controlled release [24,25,26,27].

Among amphiphilic copolymers, a group of polymerized ionic liquids (PILs) stands out, which have the form of salts due to the presence of ion pairs [28,29,30,31]. Their characteristics encompass chemical and thermal stability, high ionic conductivity, adaptable polarity providing a variety of solubility, and fitting the other properties by counterion exchange dependently on the future application [28,31,32,33,34,35,36]. In recent years, they have been investigated [37,38,39], in the formation of ionic drug conjugates [40], and polymeric micelles [41,42]. Notably, choline, as a water-soluble trimethylammonium salt with a chloride anion, is commonly employed as a naturally derived cationic constituent in biocompatible monomers generating antibacterial properties [43,44,45,46] and improved pharmacodynamic and pharmacokinetic effects of the carried drug [47]. A commercially available choline ester of methacrylic acid, [2-(methacryloyloxy)ethyl]trimethylammonium chloride (TMAMA_Cl‾), has been applied to design the choline-based PILs. Furthermore, they have been modified to the pharmaceutically active polymer conjugates by replacing chloride anion with the pharmaceutical anion, such as sulfacetamide [48], fusidate [49,50], piperacillin [51], clavulanate, and p-aminosalicylate (PAS) [37,38]. However, anion exchange has also been performed for this choline-based monomer to incorporate the pharmaceutical anion, such as cloxacillin [52], fusidate [52], salicylate [53], and PAS [54], where the resulting pharmaceutically functionalized choline monomers have been employed in the synthesis of drug-PIL ionic conjugates.

The self-assembled PILs have also been investigated as polymer matrices with the ability to encapsulate and deliver active pharmaceuticals, such as paclitaxel [55], dopamine [56], curcumin [57,58], doxorubicin [59,60], acyclovir [61,62], PAS [63], etc. In addition, PIL systems containing salicylate anions and encapsulated erythromycin have been reported to exhibit effective drug content and release [48]. In the case of dual drug systems, they are promising in combination therapy, increasing the effectiveness of treatment, for example in the presence of drug-resistant strains, as well as being more convenient because both drugs are delivered in one formulation.

**Figure 1:**
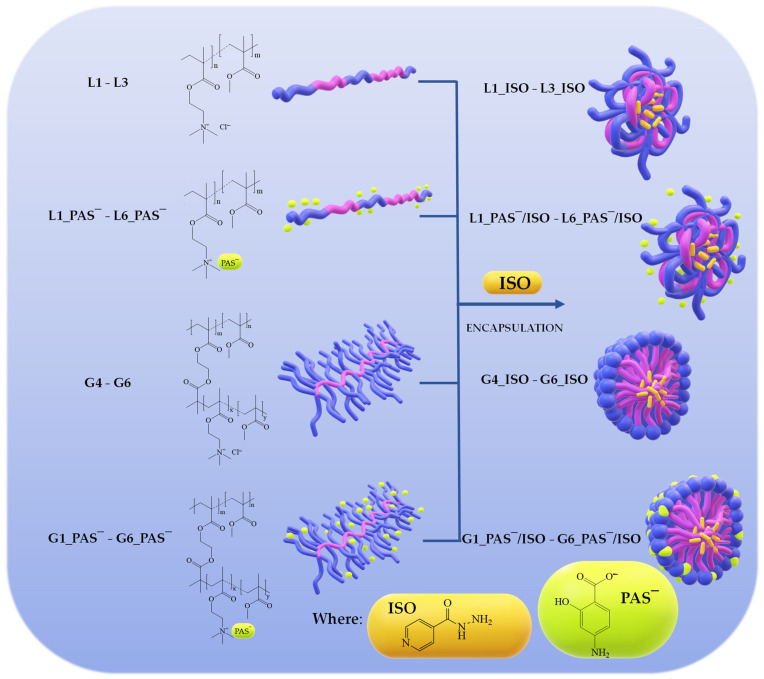
Schematic route of the linear and graft PILs to single and dual drug delivery systems combining PAS anions and encapsulated ISO.

In our studies, linear and graft choline-based PILs were investigated as matrices for single and dual drug delivery systems (Figure 1). The specific structure of copolymers led to obtaining the ionic drug conjugates, whose amphiphilic properties allowed for the encapsulation of a non-ionic drug. The polymerizable TMAMA_Cl‾ and modified [2-(methacryloyloxy)ethyl]trimethylammonium p-aminosalicylate (TMAMA_PAS‾) have been employed earlier for the synthesis of linear polymers [54,63] and graft copolymers [51,64]. Moreover, the graft copolymers based on the TMAMA_Cl‾ modified by anion exchange to obtain the PAS‾ conjugates have also been reported [51]. The amphiphilic characteristics of the selected polymers established a favorable environment for encapsulating isoniazid (ISO), which is a first-line anti-tuberculosis drug [65]. ISO exhibits strong early bactericidal activity by inhibiting the action in the synthesis of mycolic acids in Mycobacterium tuberculosis [66,67] and interindividual variability in the pharmacokinetic effects [68,69]. Furthermore, it can be used in a multidrug treatment when combined with another antituberculosis drug like the second-line PAS [70], which has the potential to extend the effective duration of ISO by retarding its acetylation path [71]. Our current research incorporated the preparation and characterization of ISO-loaded self-assembling nanocarriers based on linear and graft PILs as the single drug systems, and ionic drug conjugates as the dual drug systems carrying PAS anions and encapsulated ISO. Drug release in vitro studies were conducted in phosphate-buffered saline (PBS) simulating human body fluids at a pH of 7.4 at 37 °C to assess the potential of choline-based polymers differing in topology and composition for effective delivery of ISO and ISO/PAS.

## 2. Results and Discussion

### 2.1. TMAMA-Based Linear and Graft Copolymers as Drug Carriers

The utilization of polymerizable ionic liquids (ILs), specifically TMAMA_Cl‾ or TMAMA_PAS‾, in copolymerization with MMA via atom transfer radical polymerization (ATRP), yielded linear copolymers denoted as P(TMAMA-*co*-MMA) (Table 1) [54,63]. In this process, a low molecular weight initiator, ethyl 2-bromoisobutyrate, was applied. Moreover, graft copolymers were synthesized (Table 2) [51,64], wherein the chains of P(TMAMA-*co*-MMA) were grown by *grafting from* multifunctional macroinitiators containing 18–48% of 2-(2-bromoisobutyryloxy)ethyl methacrylate initiating units. Within each topological group of polymers, distinctions were made between polymers with Cl‾ (L1–L3 vs. G4–G6), polymers with PAS‾ directly introduced by the polymerization of pharmaceutically functionalized monomeric IL (L4_PAS‾–L6_PAS‾ vs. G1_PAS‾–G3_PAS‾), and polymers modified with PAS anions indirectly introduced by the post-polymerization exchange reaction of Cl anions in the polymer matrix using the sodium salt of PAS (L1_PAS‾–L3_PAS‾, G4_PAS‾–G6_PAS‾).

The initial ratios of comonomers TMAMA:MMA in the controlled polymerization facilitated the adjustment of the ionic content (F_TMAMA_) in the main chain of linear copolymers (18–45% of TMAMA_Cl‾ vs. 25–93% of TMAMA_PAS‾ units) or in the side chains of graft copolymers (18–39% of TMAMA_Cl‾ vs. 40–73% of TMAMA_PAS‾ units). Nevertheless, a higher content of TMAMA_PAS‾ led to the conclusion that it polymerized at a higher rate than its analog TMAMA_Cl‾, under analogical conditions. Furthermore, an increase in the number of initiating sites in MI corresponding to a higher grafting degree (DG) and the highest polymerization degree relating to the longest side chains (DP_sc_) contributed to the reduction of the ionic fraction.

The relationship between copolymer composition and the initial composition of the comonomer mixture was examined to validate the statistical structure for the majority of copolymers, as indicated by F_M1_~f_M1_, signifying comparable reactivities of comonomers (Figure S1). For copolymers L5_PAS¯, L6_PAS¯, and G2_PAS¯, G3_PAS¯, the ionic content was higher than the initial ionic monomer content in the reaction mixture (F_M1_ > f_M1_), suggesting that these conjugates can be categorized as gradient copolymers with a dominant TMAMA fraction. They facilitate the segregation of hydrophilic and hydrophobic parts, leading to a more pronounced formation of core-shell micelles compared to statistical copolymers. The nanoparticles formed by statistical copolymers result from interactions causing the shrinkage of entangled polymeric chains. In the case of graft copolymers, functioning as non-linear block copolymers, phase separation is facilitated by the main chain, generating a water-insoluble core, while side chains with either statistical or gradient structures form a layer around the core.

The introduction of PAS counterions into the polymer through the polymerization of TMAMA_PAS‾ resulted in single drug delivery systems. UV–vis analysis indicated that the drug content of PAS (DC, Table 1 and Table 2) reached 24–47% in the linear copolymers L4_PAS‾–L6_PAS‾, increasing with the ionic content, and 35–43% in the graft copolymers G1_PAS‾–G3_PAS‾, increasing with both DG and F_TMAMA_, as well as the length of side chains. In copolymers with chloride counterions, ionic exchange was beneficial for introducing the PAS counterions as pharmaceutical ones, transforming L1–L3 to L1_PAS‾–L3_PAS‾ and G4–G6 to G4_PAS‾–G6_PAS‾ with satisfactory DC levels ranging 34–51% and 32–37% [51], respectively. The highest DC was observed in sample L3_PAS‾, comparable to L6_PAS‾, corresponding to the highest F_TMAMA_ in the linear copolymer in both series. Graft copolymers G4_PAS‾–G6_PAS‾, which contained lower DP_TMAMA_ than directly obtained G1_PAS‾–G3_PAS‾ conjugates, achieved similar DCs (32–37% vs. 35–43%, respectively). These results demonstrated more controlled DC adjustment by structural parameters for copolymers derived from TMAMA_PAS‾ than in the case of post-polymerization modification of polymers yielding exchange to PAS‾. The excess chloride anions could be locally reduced by the steric hindrance of entangled polymer chains, influencing the exchange efficiency as an additional parameter.

### 2.2. Wettability and Amphiphilic Properties

Wettability measurements were conducted through goniometry using the sessile water drop method on a polymer film applied to a glass plate via spin coating. The aim was to determine the water contact angle (WCA) as a parameter for assessing the degree of hydrophilicity in the polymer systems.

**Figure 2:**
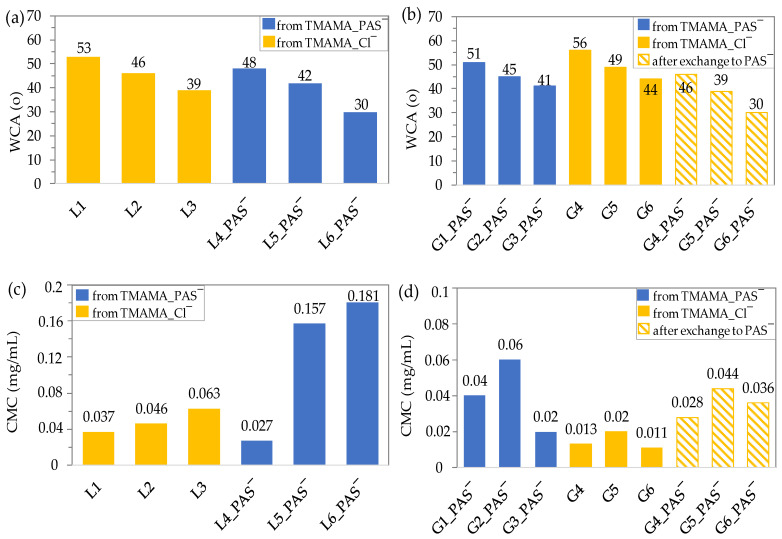
Evaluation through WCA of copolymer film spin-coated on glass plate (linear (**a**) and graft (**b**)) and CMCs of copolymers in aqueous solutions (linear (**c**) and graft (**d**)) determined by goniometry.

The results for linear copolymers showed that an increase in ionic fraction content led to a reduction in WCA (Figure 2a). For Cl-based polymers (L1–L3), this reduction ranged from 53° to 39°, while for PAS-based polymers (L4_PAS‾–L6_PAS‾), it ranged from 48° to 30° [63]. Comparing polymers containing PAS anions (L4_PAS‾) to their Cl counterparts (L2) with a similar amount of ionic fraction (~26%), the films of the latter exhibited lower surface wetting, correlated with slightly higher WCAs. This indicated reduced water interaction with the polymer surface, suggesting lower hydrophilicity.

Similarly, in the case of graft copolymers G1_PAS‾–G3_PAS‾, there was a trend of decreasing WCA with increasing F_TMAMA_, in contrast to the series of polymers G4–G6 with chloride anions (Figure 2b). Among them, G3_PAS‾ exhibited the highest wettability, characterized by loosely grafted long side chains and the highest ionic content. In Cl-contained graft copolymers, the highest grafting degree and the longest side chains in relation to the lowest content of the TMAMA fraction contributed to the high degree of hydrophilicity in copolymer G6 [51]. The exchange of Cl anions with PAS ones induced a reduction in WCA values (from 56.3–44.3° to 45.7–30.3°), resulting in films with higher wettability after introducing the drug into the polymer matrix. Moreover, these films were more hydrophilic than those in the series G1_PAS‾–G3_PAS‾ obtained by the direct incorporation of the anionic drug via PAS IL monomer, especially when compared with the densely grafted G5_PAS‾ and G6_PAS‾.

Due to amphiphilic properties, these polymers were able to form self-assembled systems in aqueous solutions, creating a conducive environment for drug encapsulation through physical interactions with the polymer matrix. To assess self-organization and drug entrapment abilities, the critical micelle concentration (CMC) was determined for both linear and graft polymers based on PILs. The CMC values were obtained through goniometry, defining the interfacial tension (IFT) of aqueous solutions of the tested polymer systems in various concentrations (c = 5 × 10^−4^–0.3 mg/mL) to identify the cross-over point in the IFT vs. logC plot (Figure S2).

For linear copolymers with chloride anions (L1–L3, F_TMAMA_ = 18–45%), CMC values ranged from 0.037 to 0.063 mg/mL. In contrast, PAS-based copolymers (L4_PAS‾–L6_PAS‾) showed a broader range of CMCs from 0.027 to 0.181 mg/mL due to higher ionic content (25–93%) (Figure 2c). An increase in CMC correlated with an elevation in the ionic fraction content was observed in both copolymer series. Comparing analogous copolymers based on Cl‾ vs. PAS‾ (L2 vs. L4_PAS‾) with similar F_TMAMA_ parameters but differences in DP_n_ and DP_TMAMA_, the latter polymer self-assembled at a lower concentration (0.046 vs. 0.027 mg/mL) due to its longer polymethacrylate chains providing lower solubility. This result emphasized the significant impact of anions within the copolymer matrix on interactions between polymer chains, thereby altering the overall self-assembly behavior.

Among graft copolymers based on TMAMA_PAS‾ IL, G3_PAS‾ exhibited the lowest CMC (0.020 mg/mL, Figure 2d), despite its high ionic content (73 mol%), with elevated DP_sc_ contributing to better stabilization of nanoparticles. Additionally, G1_PAS‾, containing a lower TMAMA fraction and a higher DP_sc_, formed nanostructures at a higher polymer concentration (0.04 mg/mL) than G3_PAS‾. The highest CMC (three times higher than G3_PAS‾), was detected for G2_PAS‾ (0.06 mg/mL), characterized by significantly short side chains. In the series of graft copolymers containing Cl‾, the highest CMC value was obtained for G5 (0.02 mg/mL), characterized by the shortest side chains and the highest ionic fraction. Two other polymers, G4 with loosely distributed chains (DG = 26%) and the highest F_TMAMA_ (39%), and G6 with densely distributed grafts (DG = 46%) and the lowest F_TMAMA_ (18%), achieved comparable CMC values (0.013 and 0.011 mg/mL, respectively). The findings allow us to conclude that the length of side chains and the arrangement of ionic groups along them exert a significant influence on the self-assembling behavior of graft copolymers and CMC values.

Comparing the series of G4–G6 with the modified G4_PAS‾–G6_PAS‾, the PAS conjugation improved polymer solubility in aqueous solution, increasing CMC values (0.013 vs. 0.028 mg/mL; 0.020 vs. 0.044 mg/mL; 0.011 vs. 0.036 mg/mL). However, these values were lower than those for G1_PAS‾–G3_PAS‾, influenced by factors such as a larger content of PAS anions (DC = 43% vs. 32%) in the case of G1_PAS‾ vs. G4_PAS‾ with similar ionic fraction content (F_TMAMA_~40%) or larger content of TMAMA fraction for G2_PAS‾ vs. G5_PAS‾ (F_TMAMA_ = 57% vs. 36%) with similar drug content (DC = 35%). The opposite correlation was observed when comparing the pair of polymers G3_PAS‾ vs. G6_PAS‾ (both with long side chains DP_sc_ = 75 vs. 65), which varied significantly by ionic fraction content (F_TMAMA_ = 73% vs. 18%) and grafting degree (DG = 18% vs. 46%). In this case, a lower density of side chains, despite the high content of ionic fraction, appeared to be beneficial for the self-assembling nanocarrier formation at lower concentrations (0.02 vs. 0.036 mg/mL, respectively).

In general, both the linear and the graft copolymers demonstrated low CMCs, classifying them as promising candidates for self-assembly behavior, favorable for the physical entrapment of drugs.

### 2.3. Encapsulation of Isoniazid

**Figure 3:**
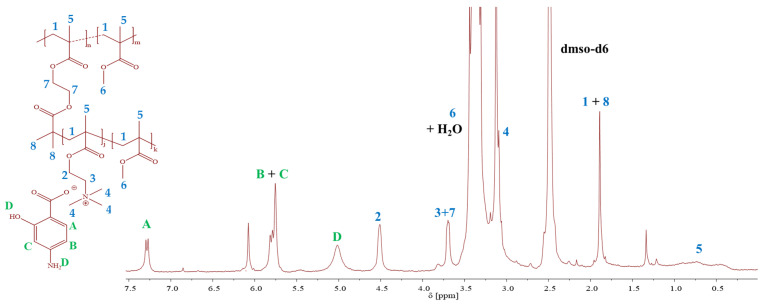
Representative ^1^H NMR spectra of the PAS conjugate based on graft copolymer with encapsulated ISO (G3_PAS‾/ISO).

Considering the self-assembling properties, all types of polymers were utilized as matrices for the encapsulation of ISO to design nanoparticles with loaded drugs, as illustrated in Figure 1. The carriers, based on the PIL copolymers with chloride anions, were pharmaceutically activated by drug encapsulation as a single drug delivery system. In turn, the PAS conjugates with loaded ISO resulted in a dual drug system. The ISO loading by copolymers was preliminarily confirmed using ^1^H NMR (Figure 3), showing in spectrum, characteristic signals related to the dispersed ISO in the matrix. These signals include protons in the aromatic ring at 7.73 and 8.7 ppm (c, b: 1H+1H, –CH), in the amide group at 10.1 ppm (a: 1H, –NH), and in the amine group at 4.5–5.3 ppm (d: 2H, –NH2). In the case of PAS conjugates, the signals of the PAS drug corresponding to the aromatic protons (B, C: 2H, 5.8–5.7 ppm) and amine (D: 2H, 5.2–4.3 ppm) were also observed.

**Figure 4:**
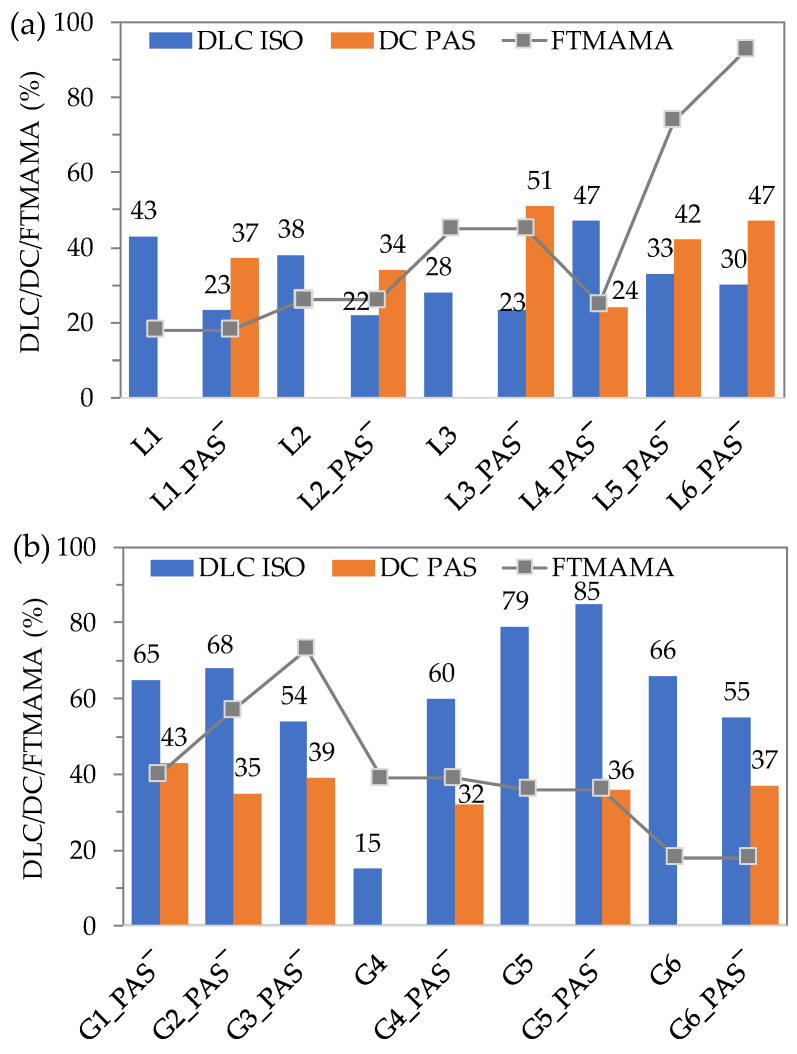
Drug content of PAS anions and drug loading content of ISO in relation to the content of ionic fraction in linear (**a**) and graft (**b**) copolymers of single and dual drug systems.

The DC of PAS in the copolymers remained consistent throughout the self-assembling process (Table 1 and Table 2, Figure 4). The drug loading content (DLC) of ISO in the copolymer nanocarriers was determined through UV–vis measurements. The DLC varied depending on the copolymer topology and composition, including the type of anions. For linear copolymers, DLC values reached 28–43% in L1–L3 and 22–23% in L1_PAS‾–L3_PAS‾, compared to 30–47% in L4_PAS‾–L6_PAS‾ (Figure 4a). These results suggest that a larger amount of TMAMA fraction with PAS counterions limited the efficiency of ISO encapsulation.

In the graft copolymer systems, the efficiency of ISO encapsulation depends on the carrier type. Generally, the process was executed with a high DLC: 54–68% for G1_PAS‾–G3_PAS‾, 15–79% for G4–G6, and 55–85% for G4_PAS‾–G6_PAS‾ (Figure 4b). Although the drug entrapment in the first series did not significantly vary, it was most successful for G2_PAS‾, characterized by a lower DP_sc_ and a higher DG, which did not hinder encapsulation. It is plausible that a high DP_TMAMA_, and consequently a high PAS content, occupied most of the space available for ISO, leading to a noticeable reduction in DLC for G3_PAS‾. For the G4 and G5 systems, the presence of PAS enhanced the ISO loading effect, especially in the case of G4 (15 vs. 60% for single vs. dual system), while in G5, DLC did not rise substantially (79 vs. 85% for single vs. dual system). The opposite relationship was observed for G6 systems, but similarly to G5, the change was not significant (66 vs. 55% for single vs. dual system). In this series of graft copolymers, both G5 systems, characterized by the lowest DP_sc_, appeared to be the most beneficial, carrying 79% of ISO (single drug system) and 85% of ISO accompanied by 36% of PAS (dual drug system).

The self-assembled conjugate systems, which are peculiar entangled polymeric chains as dual systems, present a distinctive combination of PAS and ISO drugs. They bind with the polymer matrix via ionic bonds and physical interaction, respectively. This generates different mechanisms for their release. In the case of pharmaceutical anions, the release occurs through exchange by phosphate anions present in physiological fluids, whereas the nonionic drug is removed from polymeric chains via diffusion. This specific strategy can be advantageous in the controlled delivery of both drugs, achieving an enhanced therapeutic effect.

### 2.4. Hydrodynamic Diameters of Self-Assembled Conjugates

**Figure 5:**
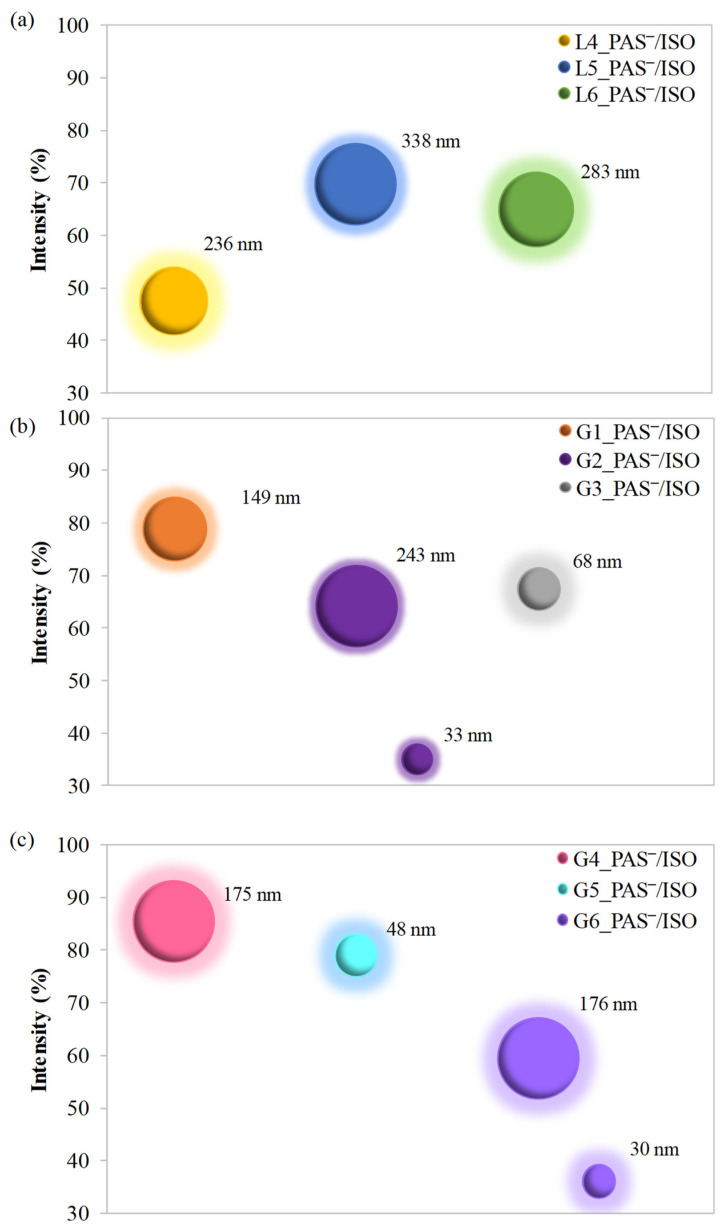
Hydrodynamic diameters (Dh) of L4-L6_PAS/ISO‾ (**a**), G1-G3_PAS/ISO‾(**b**) and G4-G6_PAS/ISO‾(**c**), ISO-loaded polymer nanoparticles determined via DLS.

The self-assembled conjugates of each group, representing dual systems bearing PAS‾ and ISO, were analyzed through DLS to evaluate the hydrodynamic diameters (Dh) of particles in water solutions. The systems mostly created two prevailing fractions, as demonstrated in Figure S3 by DLS histograms for obtained nanoparticles, whereas a schematic illustration of the predominant fractions exceeding 30% is shown in Figure 5. The linear copolymers formed nanoparticles with a size reaching 236–338 nm as the main fraction. The second fraction corresponded to smaller particles (below 50 nm), comprising a lower amount (~20%), apart from L4_PAS‾/ISO, which exhibited an equivalent fraction of dramatically larger particles with a size of 1527 nm. This phenomenon was observed due to the aggregation effect of the nanoparticles. The hydrophobic moieties in this linear system, where the fraction of hydrophilic TMAMA moieties is low (FTMAMA = 25%), formed nanoparticles through π-stacking interactions. Thus, the higher TMAMA fraction in copolymers with a linear topology has a significant impact on the lower size of formed nanocarriers.

In turn, systems based on graft copolymers formed particles with hydrodynamic diameters in the range of 149–243 nm (G1_PAS‾/ISO–G2_PAS‾/ISO) and ~175 nm (G4_PAS‾/ISO and G6_PAS‾/ISO) as the prevailing fraction, regardless of variations in DLC or polymer characteristics. The second fraction, with a smaller share, showed structures with sizes between 19 and 44 nm. However, the higher chain packing in the brushes and the high content of the TMAMA fraction in G3_PAS‾/ISO and G5_PAS‾/ISO resulted in stronger chain attraction and the formation of small nanocarriers (68 nm and 48 nm, respectively). At the same time, the aggregation effect yielded larger ones with low intensity (250 nm and 1128 nm, respectively). This occurred due to the repulsive interactions between the ionic moieties present in the side chains, especially in the systems characterized by the shortest side chains and the lowest content of TMAMA units. The polydispersity indices were below 0.5, indicating a relatively narrow size distribution of the polymer particles. The previous examination via TEM analysis of the analogical TMAMA-based graft copolymers without encapsulated drug indicated spherical geometry of the self-assembled superstructures [53], which are also assumed for the tested systems.

### 2.5. In Vitro Drug Release Studies

**Figure 6:**
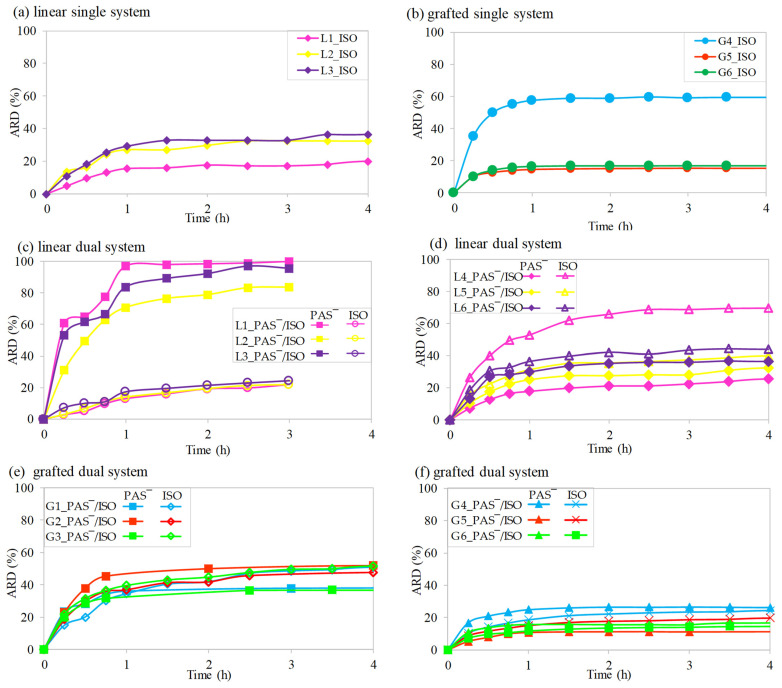
Drug release profiles for single drug systems based on copolymers with encapsulated ISO: L1–L3 (**a**), G4–G6 (**b**), and dual drug systems: L1_PAS‾/ISO–L3_PAS‾/ISO (**c**), L4_PAS‾/ISO–L6_PAS‾/ISO (**d**), G1_PAS‾/ISO–G3_PAS‾/ISO (**e**), G4_PAS‾/ISO–G6_PAS‾/ISO (**f**).

The in vitro drug release studies were conducted through dialysis under physiological conditions (pH 7.4 at 37 °C) for a duration of 72 h. For the single drug systems based on the linear copolymers, an initial burst was observed, and the encapsulated ISO was released with a relatively low efficiency of 15–29% during the first hour (Figure 6a). However, the opposite trend was noted for the linear PAS conjugates with encapsulated ISO in the dual drug systems L1_PAS‾/ISO–L3_PAS‾/ISO, resulting in fast ISO release and slow PAS release, i.e., 71–97% and 13–17%, respectively (Figure 6c). In the case of dual systems L4_PAS‾/ISO–L6_PAS‾/ISO based on conjugates obtained directly from TMAMA_PAS‾, differences in co-release of these drugs were less pronounced, but twice the amount of PAS was released compared to ISO, 31–54% vs. 18–30% (Figure 6d).

**Figure 7:**
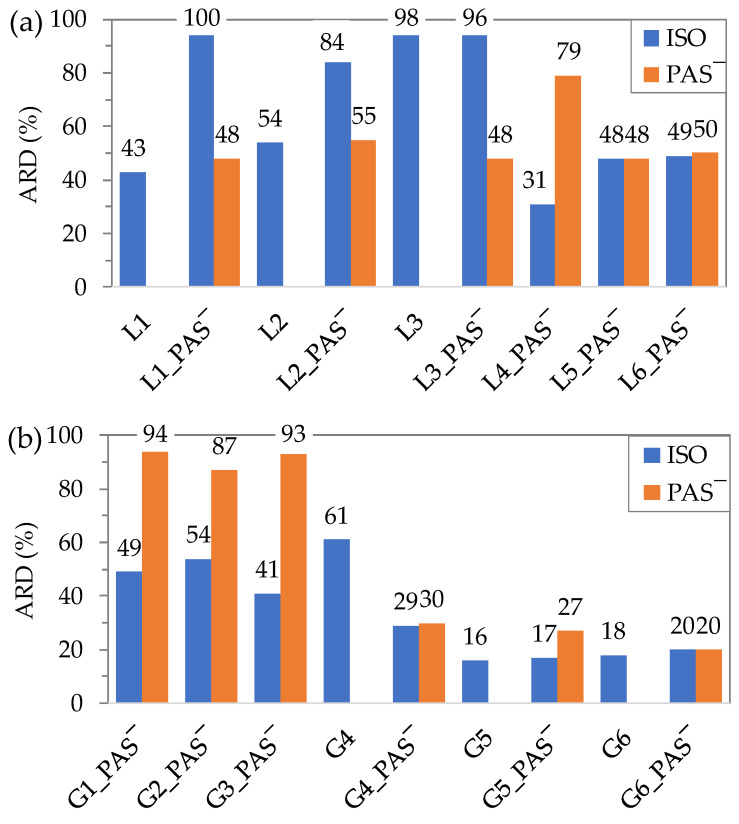
Amount of released drug by linear (**a**) and graft (**b**) copolymers of single and dual drug systems in PBS (pH 7.4 at 37 °C).

In comparison to the single L1–L3 systems, the presence of the second drug significantly influenced the co-release, enhancing ISO release while limiting PAS release. In the case of linear copolymers, the ionic chains could encapsulate the anionic drug inside the tangles, creating a hybrid interior. This means that some fraction of conjugated PAS anions could be entrapped between the polymer chains, limiting PAS release. On the other hand, ISO, physically bound to the matrix, could interact with present PAS ions, causing a repulsive effect and enhancing ISO release. The further release occurred at a slower rate with slight changes in the amounts of released drug (ARD), except for L3_ISO with the highest content of the ionic fraction, achieving complete drug release within 72 h. The final ARD values are presented in Figure 7a, showing that ISO was released in larger amounts from the single linear systems (43–98%) than in the analogous dual systems (31–100%). Additionally, the ARD of ISO was similar to the ARD of PAS (48–50%) with the exception of L4_PAS‾/ISO, where it was significantly different, yielding a release of 31% of ISO and 79% of PAS‾.

Similar to the linear copolymer systems, the release profiles for the graft copolymers generally exhibited an initial rapid logarithmic increase in ARD within the first hour, followed by a nearly linear expansion of ARD up to 72 h. The highest amounts of ISO were released within the first hour, reaching 36–46% for G1_PAS‾–G3_PAS‾ (Figure 6e), 15–60% for G4–G6 (Figure 6b), and 11–26% for G4_PAS‾–G6_PAS‾ (Figure 6f). However, subsequent drug release was limited due to the enhanced stability of the nanocarriers, as evidenced by their low CMC values. Specifically, G2_PAS‾ exhibited a faster rate of ISO release, resulting in a higher ARD compared to G3_PAS‾ with the same grafting degree but longer side chains and higher TMAMA content (54% vs. 41%, respectively). Compared to ARD from the conjugates containing only PAS prepared directly from TMAMA_PAS‾ [64], the presence of ISO marginally reduced PAS release, with G1 dropping from 100% to 94% and G2 from 91% to 87%. An exception was G3, where ARD ranged from 75% to 93% due to intensified repulsive interactions between PAS anions. Less effective release of ISO was observed for the graft copolymers G4–G6 and their PAS conjugates, with the exception of the G4 system characterized by the short backbone and lower grafting density, and simultaneously the highest TMAMA content in this series of polymers. In this single drug system, the ARD of ISO was twice as high as in its dual analog (61% vs. 29%, respectively), whereas the release profiles for G5 and G6 were comparable in the single and dual systems, yielding ~20% of ISO ARD with no impact of the ionic PAS presence. Thus, G5 was found to be the most advantageous for ISO release, indicating that the less packaging of the side chains bearing Cl‾ was favorable for drug transport, attributable to the smaller steric effect allowing superior access to the drug for release.

In the case of ionic drug release, the graft copolymers G1_PAS‾–G3_PAS‾ in dual drug systems demonstrated highly effective replacement of PAS anions by phosphate ones ranging between 87 and 94%, with a burst effect in the first hour (~50%), regardless of the polymer’s characteristics and ionic content (Figure 6e). The higher release rate of the anionic PAS in comparison to the encapsulated ISO was different from that for the polymer modified with PAS‾ to conjugates G4_PAS‾–G6_PAS‾ (Figure 6f), where ARD values of PAS and ISO were comparable. A marked discrepancy in ARD percentages emerges when contrasting the PAS conjugates obtained directly via PAS IL monomer and the PAS conjugates prepared through polymer modification via exchange of Cl to PAS (G1_PAS‾–G3_PAS‾ vs. G4_PAS‾–G6_PAS‾) (Figure 7b). The PAS release from the first series was induced by the combined influence of high F_TMAMA_ and DP_sc_ or F_TMAMA_ and DG, which amplified the repulsive forces among PAS anions. The possible partial encapsulation of PAS species during the anion exchange process in G4-, G5-, and G6-based matrices might further stabilize the nanoparticles, leading to a diminished ARD. Additionally, in these carriers, probably the PAS ions were more hidden in the polymer matrix, slowing down the drug release. It also elucidates the reduced amount of released ISO from G4_PAS‾/ISO–G6_PAS‾/ISO systems. Comparing dual systems of both types of graft copolymers, the burst release phase was prolonged from 2.5h to 4h for G4_PAS‾/ISO–G6_PAS‾/ISO.

### 2.6. In Vitro Cytotoxicity Assay

Previous reports on the cytotoxic evaluation of linear and graft copolymers based on TMAMA as matrices for drug delivery systems indicated non-cytotoxic effects towards normal human bronchial epithelial cells (BEAS-2B) and cytotoxic effects on adenocarcinomic human alveolar basal epithelial cells (A549) [72,73]. Basic cytotoxicity studies were conducted using the colorimetric MTT assay at various concentrations of polymer systems (3–100 µg/mL) on BEAS-2B cell lines to assess their impact on cell viability. For these tests, model samples were selected from the individual groups: L5_PAS‾ vs. L5_PAS‾/ISO, and G2_PAS‾/ISO vs. G6_ISO vs. G6_PAS‾/ISO. Following the administration of samples, treated cell lines were incubated for 72 h under standard conditions.

**Figure 8:**
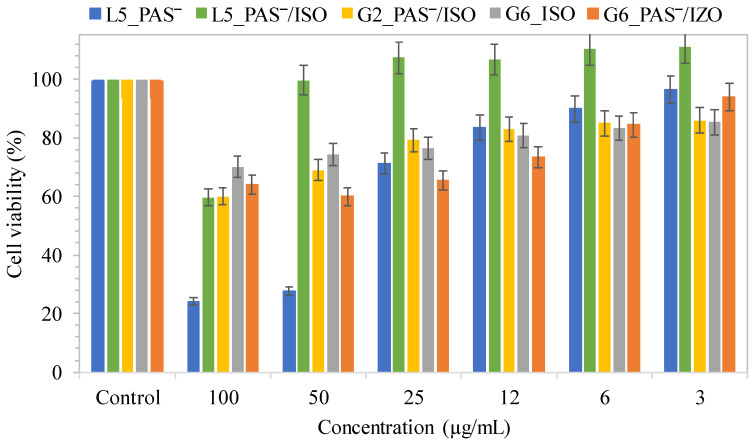
Cell viability of single and dual drug systems bearing PAS‾ or/and ISO as a selected model drug delivery systems consisted of linear or graft PIL-based copolymers at different concentrations for BEAS-2B cell line treatment, after 72 h of incubation in comparison to the controls (100%).

Cytotoxicity increased with concentration, as depicted in Figure 8. The single system with PAS‾ (L5_PAS‾) exhibited the most pronounced cytotoxic effect at the highest concentration (100 µg/mL), where cell viability decreased to 24%. In comparison to PAS conjugates based on grafted systems described previously [72], the survival rate for G6_PAS‾ equal to 54%. Interestingly, the addition of a second drug in dual systems caused a significant decrease in cytotoxicity, evident for L5_PAS‾/ISO and for G6_PAS‾/ISO at the highest concentration. Moreover, at concentrations below 100 µg/mL, L5_PAS‾/ISO did not exhibit any cytotoxicity, and its action on BEAS-2B cell lines resulted in increased cell viabilities. In contrast, the addition of the highest concentration of the single drug system G6_ISO caused the least decrease in viability, indicating that this sample is less cytotoxic at the highest tested concentration. Cell treatment with G2_PAS‾/ISO at the highest concentration reduced cell viability by up to 60%, similar to L5_PAS‾/ISO.

**Figure 9:**
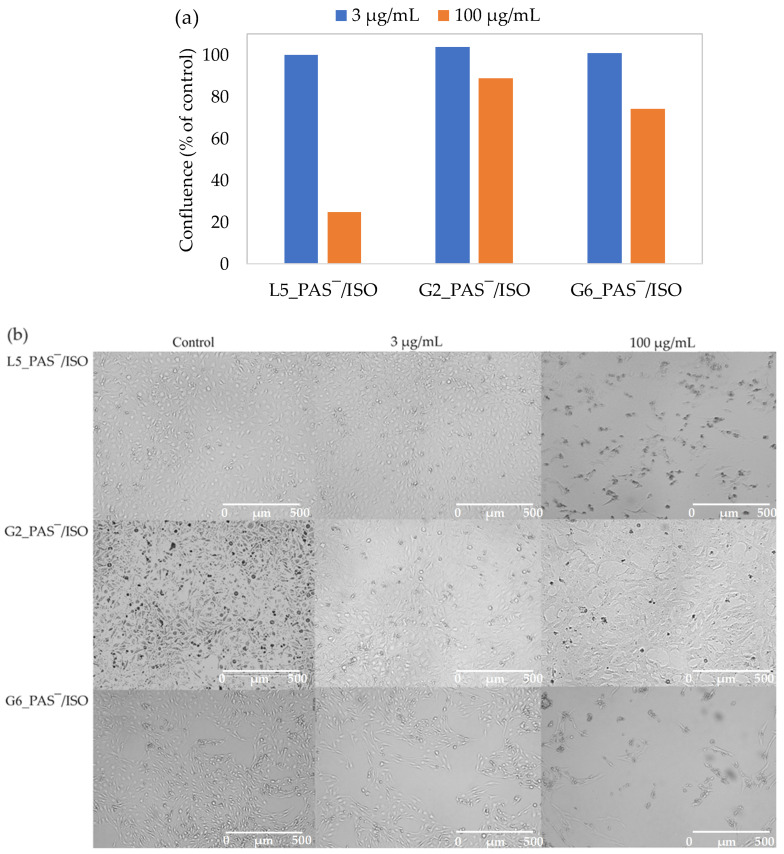
The confluence of dual systems L5_PAS‾/ISO, G2_PAS‾/ISO, and G6_PAS‾/ISO against BEAS-2B cell line (**a**), and microscopic pictures taken with a Live Cell Analyzer for untreated control cells vs. treated BEAS-2B cell lines with the dual systems L5_PAS‾/ISO, G2_PAS‾/ISO and G6_PAS‾/ISO in concentrations 3 µg/mL and 100 µg/mL (**b**).

The percentage of cell coverage, i.e., confluence, was determined after 72 h of incubation at two different concentrations of dual systems, namely 3 and 100 µg/mL. Confluence, reflecting the impact of sample treatment on BEAS-2B cell lines and influenced by cell death, was measured as a percentage relative to untreated control cells. In each sample, a comparison between the highest and lowest concentrations revealed an increase in confluence at the lowest concentration (25% vs. 98% in L5_PAS‾/ISO; 89% vs. 103% in G2_PAS‾/ISO; 73% vs. 101% in G6_PAS‾/ISO) (Figure 9a). The treatment with the L5_PAS‾/ISO system at the highest concentration led to significant cell proliferation (confluence ~25%), resulting in the smallest coverage compared to other tested systems. However, this system did not induce any harmful effects on BEAS-2B cell lines at low concentrations. Treatment with graft polymer systems at 100 µg/mL induced cell death only up to 11% and 27% in G2_PAS‾/ISO and G6_PAS‾/ISO, respectively. Microscopic pictures are presented in Figure 9b. The results indicated that these systems exhibit weak or non-cytotoxic effects against BEAS-2B cell lines, especially at low concentrations.

## 3. Materials and Methods

Linear copolymers based on TMAMA/Cl‾ (L1–L3) or TMAMA/PAS‾ (L4_PAS‾–L6_PAS‾) were synthesized as it was reported [56]. Graft copolymers based on TMAMA/PAS‾ (G1_PAS‾–G3_PAS‾) [64] or TMAMA/Cl‾ (G4–G6) [51] were synthesized by grafting from multifunctional macroinitiator. The conjugates L1_PAS‾–L3_PAS [50] and G4_PAS‾–G6_PAS‾ [51] were prepared through ionic exchange of Cl‾ in the reaction of PAS salt with polymer L1–L3 and G4–G6, respectively. Tetrahydrofuran (THF), methanol (MeOH), isoniazid (ISO), phosphate-buffered saline (PBS), DMEM-F12 medium, and 3-(4,5-dimethyl-thiazol-2-yl)-2,5-diphenyltetrazolium bromide (MTT) were received from Sigma-Aldrich (Poznań, Poland). Human bronchial epithelial cells (BEAS-2B) were purchased from ATCC (Cat# ATCC^®^ CRL-9609; Manassas, VA, USA).

### 3.1. Encapsulation of ISO

Linear or graft copolymer bearing Cl counterions, or the conjugates with PAS‾ (20 mg) and ISO (20 mg) was dissolved in methanol (2 mL). After dissolving, the deionized water (4 mL) was added dropwise into the mixture and stirred for 24 h. Then the organic solvent evaporated, whereas the aqueous fraction was lyophilized to obtain the solid product. As a result, the single drug systems bearing ISO or dual drug ones bearing PAS‾ and ISO were obtained.

### 3.2. In Vitro Drug Release Studies

Single or dual drug system (1.0 mg) was dissolved in PBS (pH = 7.4), where the concentration of the mixture was equal to 1 mg/mL. The 1 mL of the obtained solution was introduced to the dialysis membrane bag (MWCO = 3.5 kDa), which then was put into a vial filled with 44 mL of PBS. The drug release studies were carried out at a temperature of 37 °C with constant stirring. The buffer solution samples (2.5 mL) were taken to estimate the concentration of the released drug using UV–vis spectroscopy, measuring absorbance at λ = 265 nm for ISO and λ = 305 nm for PAS‾.

### 3.3. Cell Growth and Cytotoxicity Assay

Cells were grown in a DMEM-F12 medium in sterile culture bottles characterized by 75 cm^2^ of culture area, with 10% (*v/v*) FBS at 37 °C in the incubator (humidified atmosphere with 5% CO_2_). The cell cultures (10,000 cells per well) were placed in a 96-well plate for MTT tests in 0.2 mL of medium 24 h before adding polymer systems.

The MTT assay was estimated in the 96-well plates. Untreated samples were left in the first row and outer columns of wells as the controls. A series of concentrations (100–3.125 μg/mL) of the examined compounds was prepared in the remaining wells. The treated and control cells were incubated for 72 h in standard conditions. The solutions were then taken out and 50 μL of MTT solution (concentration 0.5 mg/mL in RPMI 1640 without phenol red) was added to the wells. After 2 h of incubation, the solution was removed from the wells. Formed formazan crystals were flooded and dissolved in 75 μL of isopropanol:HCl mixture (*v/v* = 1:0.04). The cytotoxicity was estimated via measurement of the formazan absorbance at 570 nm in a microplate reader. Measurements were repeated three times. The results were presented as the percentage fraction of the control. The cell viability and confluence analyses were performed with the use of a Live Cell Analyzer. After 72 h of incubation, the microscopic images were taken from treated and untreated cells.

### 3.4. Characterization

^1^H-NMR spectra were recorded with a UNITY/NOVA (Varian, Mulgrave, Victoria, Australia) spectrometer operating at 300 MHz. The measurements were carried out in deuterated dimethyl sulfoxide (DMSO) with tetramethylsilane (TMS) as an internal standard. The spectrum peaks were integrated and fitted using the MestReNova software, version: 6.0.2-5475. The critical micelle concentration (CMC) was evaluated by measuring the interfacial tension (IFT) using the pendant drop method on a goniometer (OCA 15EC, DataPhysics, Filderstadt, Germany). A series of polymers in aqueous solutions in different concentrations (0.0006–0.15 mg/mL) was prepared. A goniometer was also used for water contact angle determination (WCA), employing the sessile drop method. The polymer solution in methanol (0.3 mg/mL) was transferred by spin-coating on a thin glass plate. Next, 4 μL of deionized water was dispensed onto the polymer layer, then the WCA value was measured. The result data was processed using SCA20_U software, version: 5.0.38. The hydrodynamic diameters (Dh) of polymer particles and polydispersity index (PDI) were estimated through dynamic light scattering (DLS) using a Zetasizer Nano-S90 (Malvern Technologies, Malvern, UK). Samples in poly(methyl methacrylate) (PMMA) cells (concentration of samples = 1.0 mg/mL) were placed into the thermostatted cell compartment of the device at 25 °C. The measurements were repeated three times, to create an average value. Ultraviolet–visible light spectroscopy (UV–Vis, spectrometer Evolution 300, Thermo Fisher Scientific, Waltham, MA, USA) was used for determination of the ionic drug content (DC), the non-ionic drug loading content (DLC), as well as the amount of the released drug (ARD) during in vitro studies in PBS to estimate the absorbance of PAS‾ and ISO. Viability monitoring and confluence analysis were performed using a Live Cell Analyzer (JuLI™ Br; NanoEnTek Inc., Seoul, Republic of Korea). The cytotoxicity through the MTT test was estimated by measuring the absorbance of the formazan product at 570 nm with the use of a microplate reader (Epoch, BioTek, Winooski, VT, USA).

## 4. Conclusions

Various types of self-assembled ionic conjugates were obtained as single and dual drug systems constructed from linear and graft PIL-based copolymers. Due to the ionic structure of choline units, the copolymers could carry the drug in anionic form, i.e., PAS‾ (DC = 24–47%). Wettability assessment of chloride-based polymers indicated a hydrophilic nature (WCA = 39–56°), while the PAS conjugates exhibited higher hydrophilicity (WCA = 30–51°). The amphiphilic properties of Cl-based (CMC = 0.037–0.063 µg/mL vs. 0.011–0.020 µg/mL for linear vs. graft) and PAS-based polymers (CMC = 0.027–0.181 µg/mL vs. 0.020–0.060 µg/mL vs. 0.028–0.044 µg/mL for linear vs. graft vs. graft modified) enabled the encapsulation of ISO.

The ISO loading was sufficient in both single and dual drug systems, reaching 28–47% for linear, 54–68% for graft, and 15–85 % for graft-modified polymers. ISO was released in similar or lower amounts than PAS (16–61% vs. 20–98%, respectively). The MTT test against normal cells demonstrated low cytotoxicity of single drug systems with ISO, while the dual drug systems bearing ISO and PAS did not show any harmful action. The studies confirmed that the structure of polymers, type, and content of attached/encapsulated drugs can be used to adjust characteristics of wettability, pharmacokinetics, and cytotoxicity of systems. According to the obtained results, the designed PIL carriers, including PAS conjugates, are promising for encapsulation of ISO to attain delivery systems, including those for simultaneous co-delivery of drugs.

## Figures and Tables

**Table 1:** Basic characteristics of linear copolymers [54,63].

No.	DP_TMAMA_	DP_n_	F_TMAMA_ (mol.%)	M_n_ (g/mol)	Ð	DC (%)
**L1**	71	390	18	46,700	1.12	-
**L1_PAS‾**						37
**L2**	52	203	26	26,900	1.26	-
**L2_PAS‾**						34
**L3**	224	497	45	73,800	1.96	-
**L3_PAS‾**						51
**L4_PAS‾**	68	272	25	42,500	1.29	24
**L5_PAS‾**	139	190	74	50,300	1.33	42
**L6_PAS‾**	261	279	93	86,500	1.5500	47

Where: DP_TMAMA_—polymerization degree of TMAMA units; DP_n_—total polymerization degree; F_TMAMA_—the content of TMAMA units in the copolymer; M_n_—average molecular weight; Ð—dispersity index; DC—drug content of PAS‾.

**Table 2:** Basic characteristics of graft copolymers [51,64].

No.	n_sc_	DG (%)	DP_sc_	DP_TMAMA_	F_TMAMA_ (mol.%)	M_n_ (g/mol)	Ð	DC (%)
**G1_PAS‾**	99	48	89	35	40	841,900	1.40	43
**G2_PAS‾**	65	18	35	20	57	431,300	1.36	35
**G3_PAS‾**			75	55	73	405,000	1.46	39
**G4**	48	26	35	15	39	273,100	1.15	-
**G4_PAS‾**								32
**G5**	133	46	28	11	36	583,500	1.03	-
**G5_PAS‾**								36
**G6**			65	12	18	1,090,500	1.11	-
**G6_PAS‾**								37

Where: n_sc_—the number of the side chains; DG—the degree of grafting, equal to n_sc_ per polymerization degree of the main chain; DP_sc_—the polymerization degree of the side chains, F_TMAMA_—content of TMAMA in the side chains; M_n_—average molecular weight; Ð—dispersity index; DC—drug content of PAS‾.

## Data Availability

Data are contained within the article and Supplementary Materials.

## Supplementary Materials

The supporting information can be downloaded at https://www.mdpi.com/article/10.3390/ijms25021292/s1.
