# Supplementary material for: Ionic Liquid-Based Polymer Matrices for Single and Dual Drug Delivery: Impact of Structural Topology on Characteristics and In Vitro Delivery Efficiency
Source: Int J Mol Sci. 2024 Jan 20;25(2):1292. doi: 10.3390/ijms25021292 (PMC10816880; doi:10.3390/ijms25021292)
Supplement: Supplementary file 1 [file ijms-25-01292-s001.zip › ijms-2797518-supplementary.pdf]

Supplementary Materials

Ionic Liquid based Polymer Matrices for Single and Dual Drug Delivery: Impact of Structural Topology on Characteristics and In Vitro Delivery Efficiency

Katarzyna Niesyto [1], Shadi Keihankhadiv [1], Aleksy Mazur [1], Anna Mielańczyk [1], Dorota Neugebauer [1]

[1] Department of Physical Chemistry and Technology of Polymers, Faculty of Chemistry, Silesian University of Technology, 44-100 Gliwice, Poland; Katarzyna.Niesyto@polsl.pl (K.N.); Shadi.Keihankhadiv@polsl.pl (S.K.); Aleksy.Mazur@polsl.pl (A.Ma.), Anna.Mielanczyk@polsl.pl (A.Mi.)
* Correspondence: Dorota.Neugebauer@polsl.pl (D.N.)

Content:

Figure S1. Copolymer composition vs. initial composition of the comonomer mixture.

Figure S2. Exemplary variation of the surface tension with the logarithm of the concentration of linear copolymer (a) L3, and grafted copolymers (b) G5 and (c) G5_PAS^{-} conjugate in aqueous solution at 25°C.

Figure S3. DLS histograms for nanoparticles based on ISO loaded (a) linear copolymers, (b) graft copolymers prepared from TMAMA_PAS, and (c) modified graft copolymers prepared from TMAMA_Cl as dual drug systems in deionized water at 25 °C.

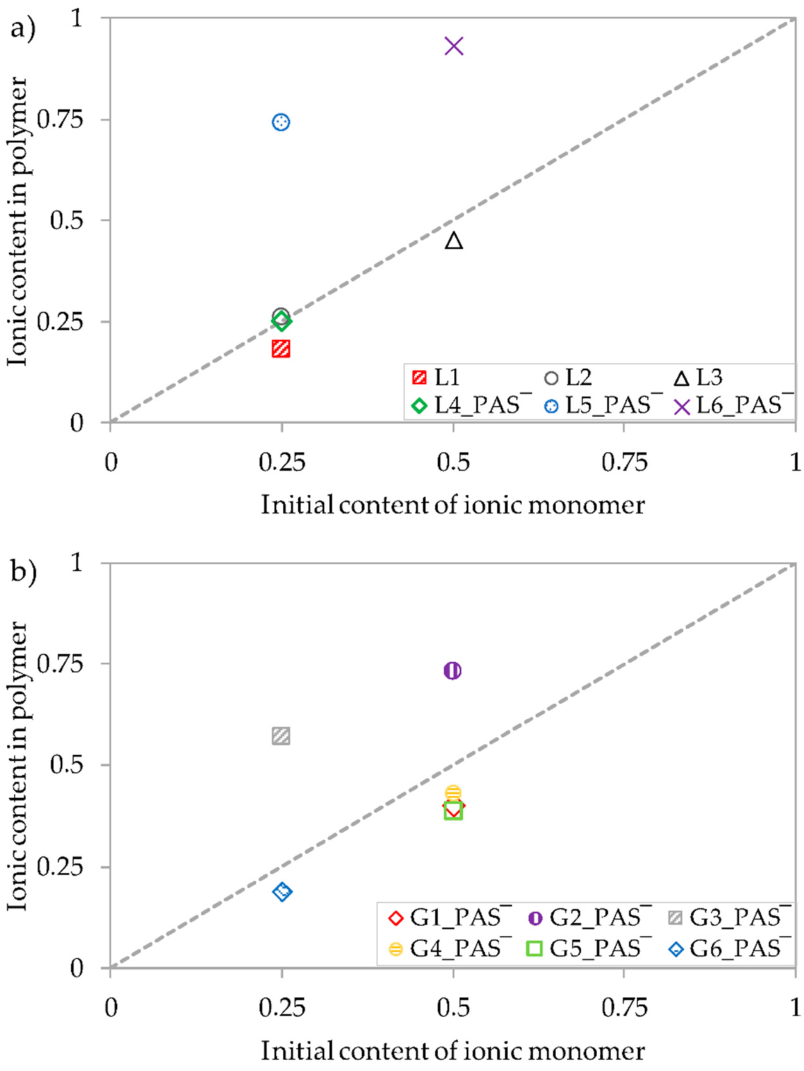

Figure S1. Copolymer composition vs. initial composition of the comonomer mixture.

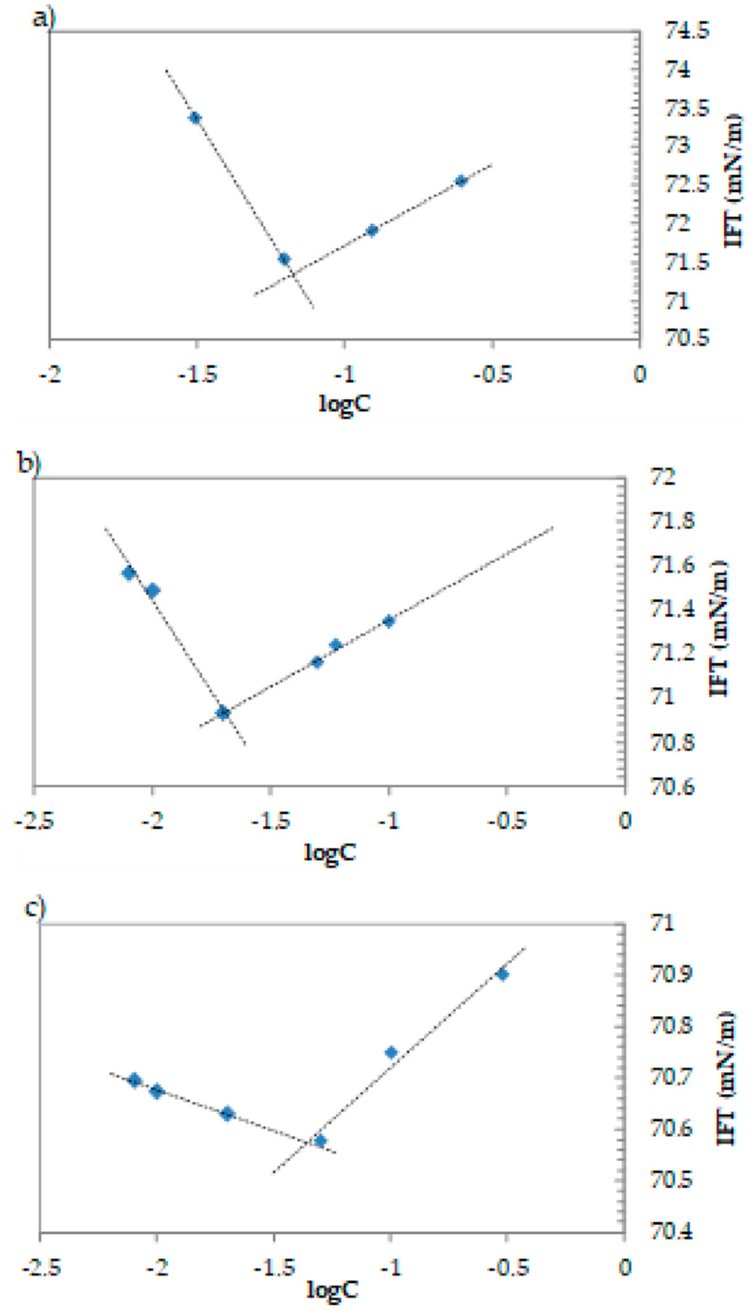

Figure S2. Exemplary variation of the surface tension with the logarithm of the concentration of linear copolymer (a) L3, and grafted copolymers (b) G5 and (c) G5_PAS^{-} conjugate in aqueous solution at 25°C.

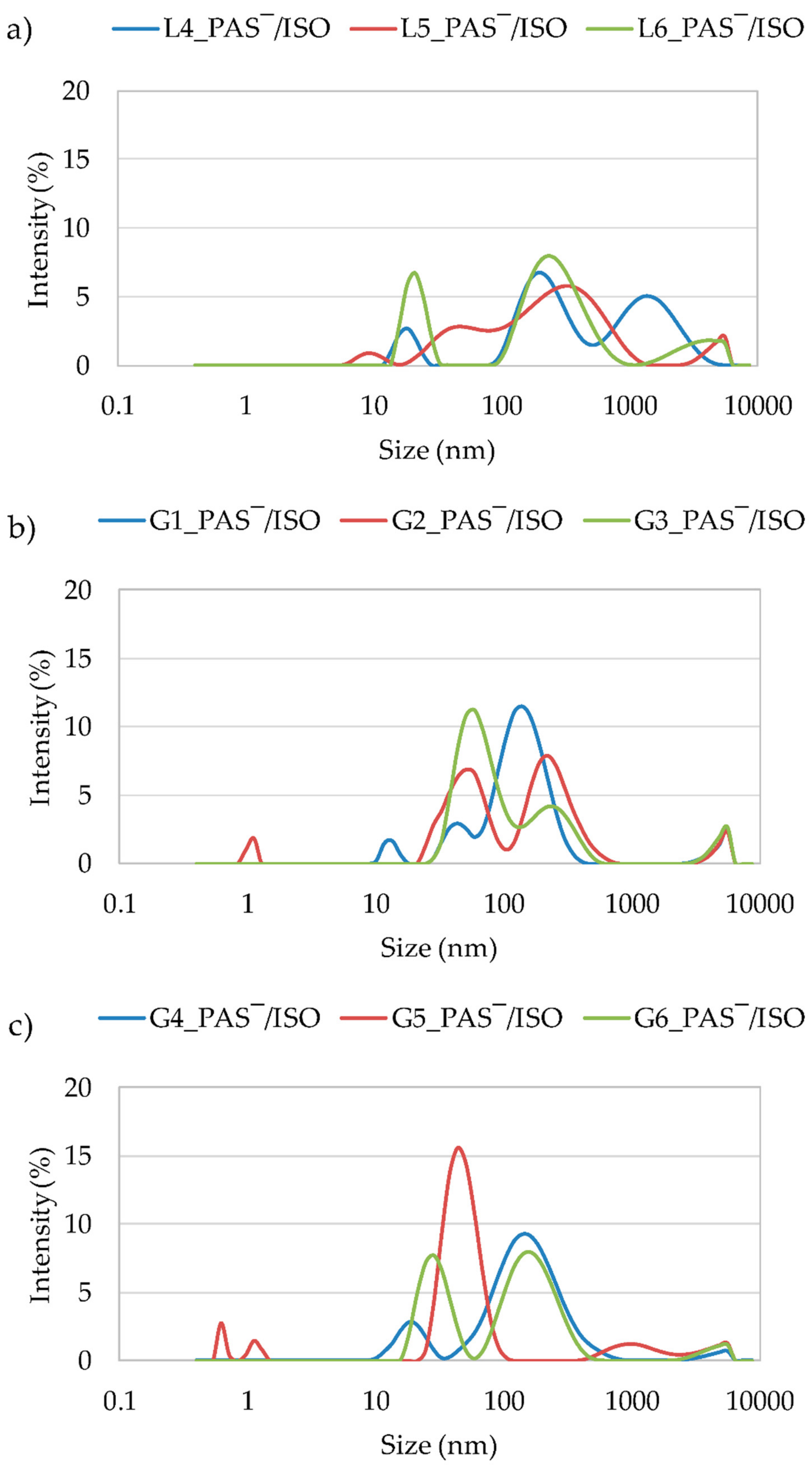

Figure S3. DLS histograms for nanoparticles based on ISO loaded (a) linear copolymers, (b) graft copolymers prepared from TMAMA_PAS, and (c) modified graft copolymers prepared from TMAMA_Cl as dual drug systems in deionized water at 25 °C.
